# On the need for epidemiology in psychiatric sciences

**DOI:** 10.1017/S2045796019000507

**Published:** 2019-11-25

**Authors:** Corrado Barbui, Oye Gureje, Scott B. Patten, Bernd Puschner, Graham Thornicroft

**Affiliations:** 1 World Health Organization Collaborating Centre for Research and Training in Mental Health and Service Evaluation, Department of Neuroscience, Biomedicine and Movement Sciences, Section of Psychiatry, University of Verona, Verona, Italy; 2 World Health Organization Collaborating Centre for Research and Training in Mental Health, Neurosciences and Substance Abuse, Department of Psychiatry, College of Medicine, University of Ibadan, Ibadan, Nigeria; 3 Department of Community Health Sciences, Cumming School of Medicine, University of Calgary, Canada; Department of Community Health Sciences, Department of Psychiatry, Cumming School of Medicine, University of Calgary, Canada; 4 Department of Psychiatry II, Ulm University, Ulm, Germany; 5 Centre for Global Mental Health and Centre for Implementation Science, Institute of Psychiatry, Psychology, and Neuroscience, King's College London, London, UK

**Keywords:** Epidemiology, health outcomes, health service research, mental health, psychiatric services

Epidemiology and Psychiatric Sciences is an international, peer-reviewed journal established in 1992 by Michele Tansella (Thornicroft, 2017). It was originally intended to promote the use of an epidemiological approach to the study of all aspects related to the promotion of mental health, and to the prevention and treatment of mental disorders. The first Editorial, published in Italian in the first issue in 1992, emphasised the need to assist the growth of epidemiological psychiatry as a new discipline, based on the use of epidemiological methods to advance knowledge in the field of social psychiatry and, more generally, in all the diverse fields of psychiatric sciences (Tansella, 1992). In that visionary Editorial Michele Tansella quoted John Ziman, who wrote in 1976: ‘*…the hallmark of a new discipline is the establishment of a specialised journal catering to the scholarly needs of its exponents. It constitutes an act of solidarity and sodality, and polarises the subject around it*’ (Ziman, 1976). The journal was launched as *Epidemiologia e Psichiatria Sociale* initially, and renamed as Epidemiology and Psychiatric Sciences in 2010 when it turned into an international journal with contributions in English only. The cover was green to convey a sense of hope, health, adventure and renewal, as well as self-control, compassion and harmony.

After more than 25 years Epidemiology and Psychiatric Sciences still has a green (although virtual) cover, has published hundreds of high-quality original contributions, editorials, commentaries and special articles, and from January 2020 has moved to an open access format. It continues to strive to promote the use of epidemiology to advance knowledge in mental health. Our vision is that epidemiology represents a unique opportunity for describing and understanding reality, and for monitoring and critically evaluating the actions and initiatives aimed at improving mental health in the real world.

Pragmatically, Epidemiology and Psychiatric Sciences calls for papers in the field of mental health *promotion*. Mental health promotion involves actions that support people to adopt and maintain healthy lifestyles and which may create supportive living conditions or environments for mental health. It includes advocacy and policy actions (Naslund *et al*., 2016; Lempp *et al*., 2018), legislative and regulatory reforms (Chisholm *et al*., 2017) and research and evaluation projects in settings at all stages of economic development (de Vries *et al*., 2018). Epidemiology and Psychiatric Sciences has been contributing to the progressive growth of the evidence base needed to determine the cost-effectiveness of such initiatives and actions.

Epidemiology and Psychiatric Sciences also calls for papers in the field of *prevention* of mental disorders. Given the current limited evidence of the effectiveness of treatment modalities for decreasing disability due to mental disorders, a key sustainable method for reducing the burden caused by these disorders is prevention. As many common mental disorders are shaped to a great extent by the social, economic and physical environments in which people live (Purgato *et al*., 2017; Inoue *et al*., 2019), addressing these social, economic and physical factors at both population and individual levels can be expected to improve the health of the world's population, reducing inequalities and preventing the development of mental disorders. We need to rapidly expand the evidence base on effective actions that can be implemented in countries at all stages of economic and social development (World Health Organization and Gulbenkian Foundation, 2014).

Epidemiology and Psychiatric Sciences in addition calls for papers in the field of *treatment* of mental disorders, as the efficacy and safety of pharmacological and non-pharmacological treatments is far from optimal, functional and quality of life outcomes are rarely considered, and the long-term beneficial and harmful consequences of interventions are almost never assessed. Studies covering the whole pathway between the generation of evidence to its uptake in routine practice should be conducted, ideally in settings at different stages of development. Epidemiology and Psychiatric Sciences calls for papers reporting the results of randomised trials, including pragmatic randomised studies, systematic reviews and meta-analyses, evidence-based guidelines and implementation studies (Chung *et al*., 2018; Koike *et al*., 2018; Solmi *et al*., 2018; Tomlinson *et al*., 2018; Khan *et al*., 2019; Turrini *et al*., 2019). As mental health care is usually composed of a combination of diverse psychological, pharmacological and social interventions, delivered within human and therapeutic relationships in a given context of care, complex multifaceted interventions should also be given due consideration, including studies monitoring the performance of mental health services and assessing the capacity of mental health systems for providing access to optimal care to those in need (Barbui *et al*., 2017). Epidemiological analyses of existing large databases are additionally warranted to generate knowledge on the real-world use and outcome of a variety of mental health interventions.

The new open access format of the Journal has both advantages and drawbacks. The main advantage is that readers, anywhere in the world, will be able to access any type of published contribution without a subscription. This is a major development, fully in line with current policies on open science and data sharing (Barbui *et al*., 2016), and aligned with the journal's mission of promoting the use of epidemiological methods in mental health. The main disadvantage, however, is that in place of subscriptions, authors will be asked to pay an article processing charge. However, this disadvantage will be mitigated by the following factors: (1) the journal offers 100% automatic waivers or a 50% discount for papers where the corresponding author is based in a low-resource country, depending on a pre-defined set of economic indicators (https://www.research4life.org/access/eligibility/); 2) authors will have the opportunity to apply for discretionary waivers and 3) authors of invited materials, including editorials and special articles, will not be asked to pay the article processing charge, meaning that the journal Editors will continue to be free to implement a very well-oriented editorial agenda, in line with the themes briefly mentioned in this Editorial, always attempting to shed light on important and controversial topics that do not receive enough attention globally.

Based on these premises, our intention for the future is that the new open access version of Epidemiology and Psychiatric Sciences will be able to fulfil its role more powerfully, energising and consolidating the discipline of epidemiological psychiatry around its scientific community, being responsive and open to the needs of its exponents, including professionals, policy makers, politicians, service users and their families and the wider society.
